# Optimization of Immune Checkpoint Blockade via a Multiscale Model System

**DOI:** 10.1002/cso2.70007

**Published:** 2025-11-28

**Authors:** Anne M. Talkington, Anthony J. Kearsley

**Affiliations:** ^1^ Applied and Computational Mathematics Division National Institute of Standards and Technology Gaithersburg Maryland USA; ^2^ Division of Pharmacokinetics Pharmacodynamics, and Systems Pharmacology, Department of Pharmaceutical Sciences University at Buffalo Buffalo New York USA

**Keywords:** agent‐based model, dynamical system, immune checkpoint blockade, immune exhaustion

## Abstract

Cancer progresses when cancer cells selectively bind to inhibitory receptors on a T cell surface, downregulating tumor immune response. One standard‐of‐care strategy to combat this process is immune checkpoint blockade. Immune checkpoint blockade occurs when a therapeutic agent binds to, and inhibits, inhibitory receptors on a T cell surface, such that immune stimulation is favored when T cells and cancer cells interact. However, many cancers fail to respond to immune checkpoint blockade treatments. Here we explore a whole‐tumor and an individual cell‐focused model system to test expected outcomes of blockade perturbations in tumor‐immune interactions. We first observe a transition point at which patients become more likely to reach “remission” or “stable disease” as a terminal state, and a “progressive disease” state is less likely. We propose a physical, agent‐based framework for testing blockade strategies at the cellular level. This offers valuable guidance for blockade efficacy optimization in future development and design of therapeutic antibodies.

## 1 Introduction

Cancer cells avoid immune recognition by leveraging the immune checkpoint system [1]. Cancer cell surface receptors bind to inhibitory T cell surface receptors, including programmed cell death protein 1 (PD‐1) and cytotoxic T‐lymphocyte associated protein 4 (CTLA‐4) [2, 3, 4, 5], with higher affinity than stimulatory receptors [6]. To oppose and “blockade” this mechanism, standard‐of‐care treatments include administration of anti‐PD‐1 or anti‐CTLA‐4 antibodies [7, 8]. Systemic immune checkpoint blockades are often a preferred treatment strategy for advanced cancers, particularly melanoma and non‐small cell lung cancer (NSCLC) [9, 10, 11, 12]; however, their success rates are highly variable [13]. Specifically, response rates are reported as to 25% to 45% in melanoma and 8% to 40% in NSCLC, indicating room for improvement in this strategy [14, 15, 16, 17].

This problem can be framed as receptor competition to control immune activity. Inhibitory receptors are advantaged. For example, the dissociation constant (Kd) values for stimulatory interactions between CD28 and CD80/CD86 are 4 µmol.L^−1^ or 20 µmol.L^−1^, respectively [6]. The inhibitory receptor CTLA‐4 binds to CD80 with a Kd of 0.2 µmol.L^−1^ to 1.4 µmol.L^−1^ and CD86 with a Kd of 2.6 µmol.L^−1^ to 22.4 µmol.L^−1^ [6], suggesting an order of magnitude difference in affinity between these receptors associated with stimulating versus exhausting interactions.

Dynamical systems have a rich history in mathematical oncology as a method for framing cancer growth and interactions with immune cells [18, 19, 20, 21, 22]. Ordinary differential equation (ODE) models allow for analytic and numerical methods to test steady states corresponding with terminal disease outcomes, given parameters and initial conditions for a patient's tumor and therapeutic intervention. Combining dynamical systems approaches with an agent‐based model (ABM) allows for the examination of entire tumor‐level behaviors and stochastic cell‐level behaviors.

A dynamical systems approach to modeling tumor‐immune interactions is demonstrated wherein a perturbation simulating immune checkpoint blockade is introduced. The results inform a complementary agent‐based model for individual cell behaviors during simulated treatment. This approach predicts and tests the desired efficacy of immune checkpoint blockade in silico.

## 2 Methods

### 2.1 Dynamical Systems Derivation and Simulation

We simulated tumor‐immune interactions according to nonlinear ODEs following those explored by Kuznetsov et al. [23] and Talkington et al. [24] (Equations (1), (2), (3), (4)),

(1) $$\frac{dT}{dt}=b_{T}+\frac{r_{1}TC}{k_{1}+C}-a\left(A+C\right)T-d_{T}T,$$

(2) $$\frac{dC}{dt}=b_{C}C\left(1-\frac{C}{k_{2}}\right)-r_{2}CT-d_{C}C,$$

(3) $$\frac{dA}{dT}=b_{A}-d_{A}A,$$

where

(4) $$a=\frac{1}{n}IR_{1}R_{2}$$

Effector T cell count is denoted by *T, C* represents cancer cell count, and *A* represents non‐cancer antigen‐presenting cell (APC) count. Cell types are modeled with modified birth‐death processes. T cells are stimulated via a Michaelis–Menten inspired rule for receptor availability and saturation. When their inhibitory receptors are engaged by APCs, T cells become exhausted. Cancer cell growth is modeled according to a logistic rule accounting for nutrient limitations and physical constraints. Cancer cells are killed when engaged with T cells according to the effector T cell killing rate. The exhaustion rate coefficient *a* is derived from the principles of mass action, which has previously been used to represent the necessary engagement of available receptors (*R*_1_ and *R*_2_) on interacting cells [25, 26, 27]. It is further modified by the number of stimuli (*n*) required for exhaustion, to represent the stochastic robustness of the T cells in each simulated tumor [28], as well as the blockade efficiency control *I*.

Initial conditions and parameter values (Table 1) were selected according to mouse data [23, 24]. Model iterations were conducted with Monte Carlo sampling from a normal distribution around literature‐derived parameter values. Standard deviations were initially set to 10% of the mean and adjusted such that sufficient sampling would be achieved around small parameters, while large variances would not dominate the search space. This gave us a starting point for our search, though parameter spaces were further adjusted to remove non‐biological outcomes, for example, negative cell counts. The iterative sampling process corresponded to a virtual population where each parameter set represented a tumor. Negative or close‐to‐zero sampled parameters were set to 1e‐10. Immune checkpoint blockade values were sampled between (0,1). A value of *I* = 0 represented a perfect blockade (no exhausting interactions); a value of *I* = 1 represented no blockade (all exhausting interactions).

**TABLE 1:** Parameters in the ODE model system.

Parameter	Interpretation	Units	Sampled distributions
*b*_T_	T cell “birth” rate	cells day^−1^	*N*(13,000, 100^2)
*b*_C_	Cancer cell “birth” rate	day^−1^	*N*(0.18, 0.01^2)
*b*_A_	Non‐cancer APC “birth” rate	cells day^−1^	*N*(10,000, 100^2)
*d*_T_	T cell “death” rate	day^−1^	*N*(0.0412, 0.01^2)
*d*_C_	Cancer cell “death” rate	day^−1^	*N*(1.8e‐10, (1e‐11)^2)
*d*_A_	Non‐cancer APC “death” rate	day^−1^	*N*(0.0412, 0.01^2)
*r*_1_	Rate of T cell stimulation	day^−1^	*N*(0.1245, 0.01^2)
*r*_2_	Rate of cancer cell killing	day^−1^ cells^−1^	*N*(1.101e‐7, (1e‐8)^2)
*k*_1_	Saturation of T cell stimulation	cells	*N*(2.019e7, 1000^2)
*k*_2_	Cancer cell carrying capacity	cells	*N*(2e9, 10,000^2)
*R*_1_	Receptor concentration—stimulating cell	cells^−1^	*N*(sqrt(3.422e‐5), (1e‐3)^2)
*R*_2_	Receptor concentration—stimulated cell	cells^−1^	*N*(sqrt(3.422e‐5), (1e‐3)^2)
*n*	Minimum number of interactions for exhaustion phenotype	cells	*N*(10,000, 1000^2)
*I*	Immune checkpoint blockade efficiency	cells^2^ day^−1^	U(0, 1)

The model was nondimensionalized (Equations (5), (6), (7)) by scaling time and cell number according to the methods in [23, 24], reducing the parameter spread. Nondimensional parameters were calculated from original parameter values, and new values were Monte Carlo‐sampled from a normal distribution around each of the nondimensional parameters,

(5) $$\frac{dx}{d\tau }=\sigma +\frac{\rho xy}{\eta +y}-\mu \left(z+y\right)x-\delta_{T}x,$$

(6) $$\frac{dy}{d\tau }=\alpha y\left(1-\frac{y}{\beta }\right)-xy-\delta_{C}y,$$

(7) $$\frac{dz}{d\tau }=\gamma -\delta_{A}z.$$

Sensitivity analyses were performed for parameters in the nondimensionalized system. We performed both a global analysis, with partial rank correlation coefficients (PRCC) [29], and a local analysis, by sweeping though parameter space subsets. Simulations were performed with ode15s in MATLAB (2024a).

### 2.2 Agent‐Based Modeling (ABM) Approach

**FIGURE 1:**
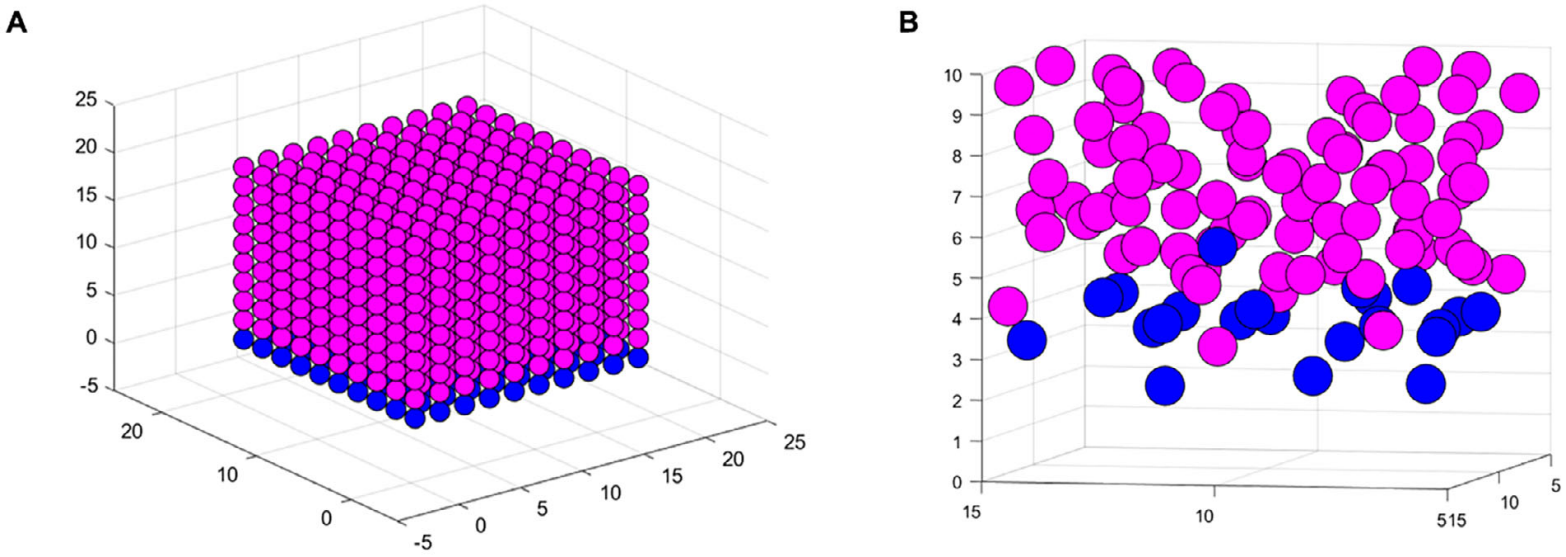
ABM representation of solid tumor. (A) Initialization of tumor cell grid (magenta) and T cell layer (blue). (B) Zoomed‐in screen capture of cell positions illustrating T cell infiltration in the cancer cell layers and tumor expansion due to mechanical forces (time step = 300).

Immune checkpoint blockade was simulated in a virtual, small tumor microenvironment by initializing a 10 × 10 × 10 grid of cells. Each cell was represented by a sphere of radius and mass 1 (Figure 1A). To simulate T cell infiltration, we defined the grid's bottom layer as T cells with initial *y*‐ and *z*‐velocities. Diagonal initialization proved the most successful for infiltration (Figure 1B), consistent with “rolling” T cells breaching the vascular endothelium [30, 31]. A T cell forcing term, representative of cytokine and chemokine signaling [32], was prescribed. Cancer cells were given random initial perturbations. Physical interactions between cells were modeled according to a Lennard–Jones potential where *r* was the distance between the centers of each pair of cells, and *ε* = *σ* = 1 was assumed [33, 34, 35]. Though traditionally applied to molecular interactions, Lennard–Jones models have successfully been implemented to describe interactions at the cellular level [33, 34, 36, 37]. The Verlet algorithm [38] advanced the model through time (*dt* = 0.01). Open boundary conditions were used, and a tendency of the tumor to expand was noted, consistent with mechanical forces in part driving metastatic potential [39]. At each time step, interactions were tallied between pairs of cells in physical contact. Exhausting interactions were predicted according to a blockade efficiency, that is, a probability of exhaustion per interaction, that defined each T cell's trajectory. Simulations were performed using Python (3.11.7) and post‐processed in MATLAB (2024a).

## 3 Results and Discussion

### 3.1 Dynamical Systems Results and Discussion

**FIGURE 2:**
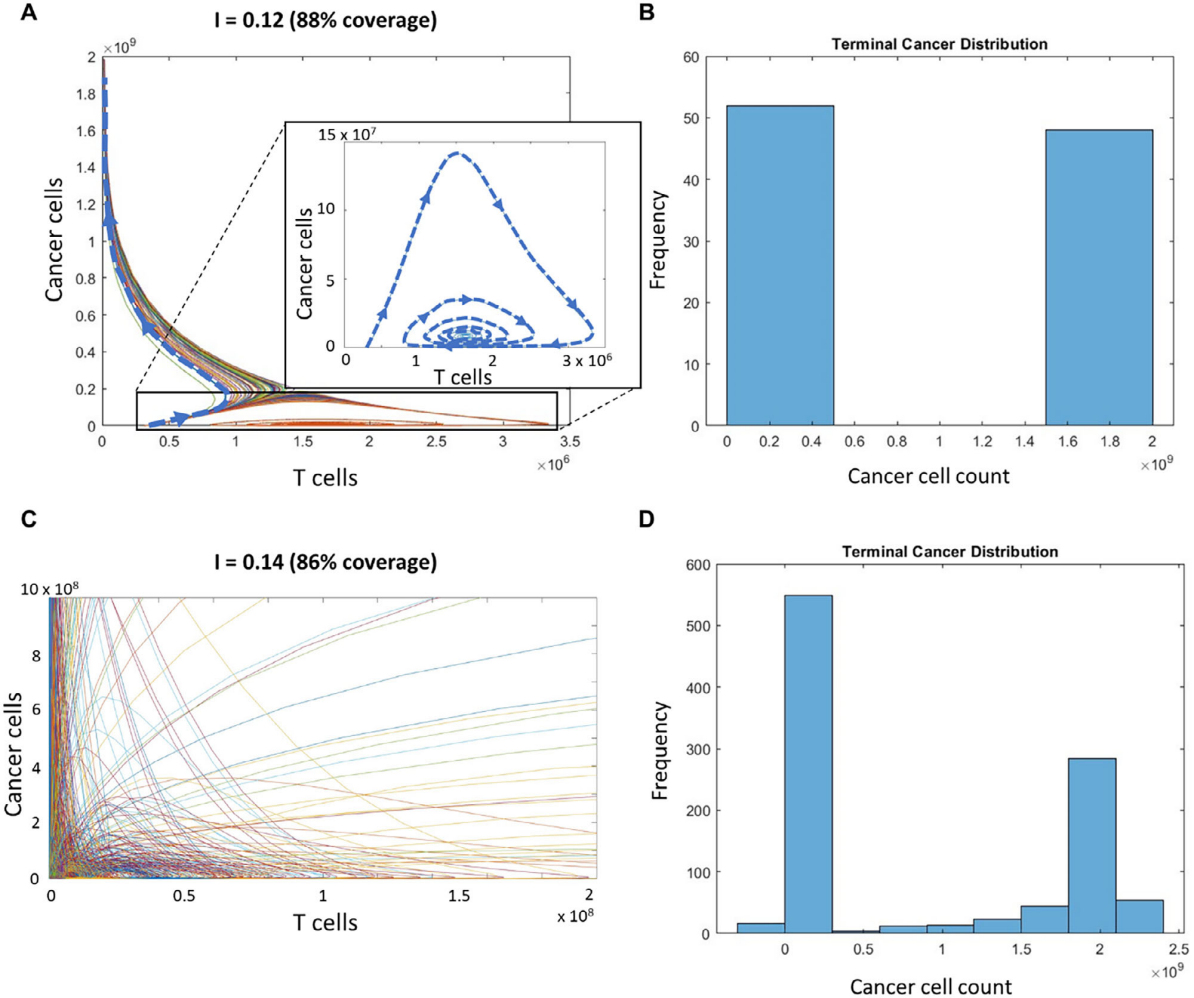
ODE simulation for tumor‐immune dynamics. (A) Phase plane representing cancer cell vs. T cell count in the simulated tumor, sweeping through *I* (*n* = 100 runs). Each trajectory represents one tumor. Dashed lines with arrows trace one trajectory illustrative of each fate—progressive disease (high cancer, low T cell; left region of the plane) or immune control (high T cell, low cancer; bottom region of the plane magnified in callout). (B) Terminal cancer cell count from the simulation in (A) illustrating progressive disease or stable disease as a binary outcome. (C) Phase plane representing cancer cell vs. T cell count in the simulated tumor, sweeping through *I* with randomized parameters (*n* = 1000 runs). Each trajectory represents one tumor. While their placement in the plane is more variable, trajectories follow one of the two progressions to progressive disease or immune control highlighted in panel (A). (D) Terminal cancer cell count from the simulation in (C) illustrating progressive disease or stable disease as a distribution.

Sweeping through the parameter *I* yielded trajectories for two distinct steady states in the cancer versus T cell phase plane (Figure 2A, B). We first swept through *I* exclusively and observed a binary terminal distribution of cancer cells, where the percent of runs ending in the “progressive disease” (high cancer, low T cell) versus “stable disease” (T cells controlling cancer cells) state depended on *I*. When *I* represented 80% to 90% blockade, we observed a transition such that patients were less likely to reach progressive disease and more likely to reach the immune‐controlled state (Figure 2A, B). This transition point was defined at the value of *I* for which 50% of the simulated tumors ended in each steady state. Our predicted blockade efficiency values are consistent with values explored in previous computational and experimental work (60% to 90% occupancy of PD‐1 receptors) [40], supporting the feasibility of such a strategy.

These trends remained when the sweep through *I* was repeated and all parameters were varied according to Monte Carlo sampling (Table 1), accounting for population heterogeneity. This simulation produced a distribution of tumor sizes rather than a binary “tumor”/“no tumor” endpoint, although the pattern of trajectories towards either “progressive disease” or “stable disease” steady states remained unchanged (Figure 2C, D). We observed shifts in the separatrix with some parameter combinations, consistent with the relative difficulty of treating tumors in which a larger regime leads to progressive disease. However, these tumors maintained some ability to reach stable disease via immune checkpoint blockade perturbations.

**FIGURE 3:**
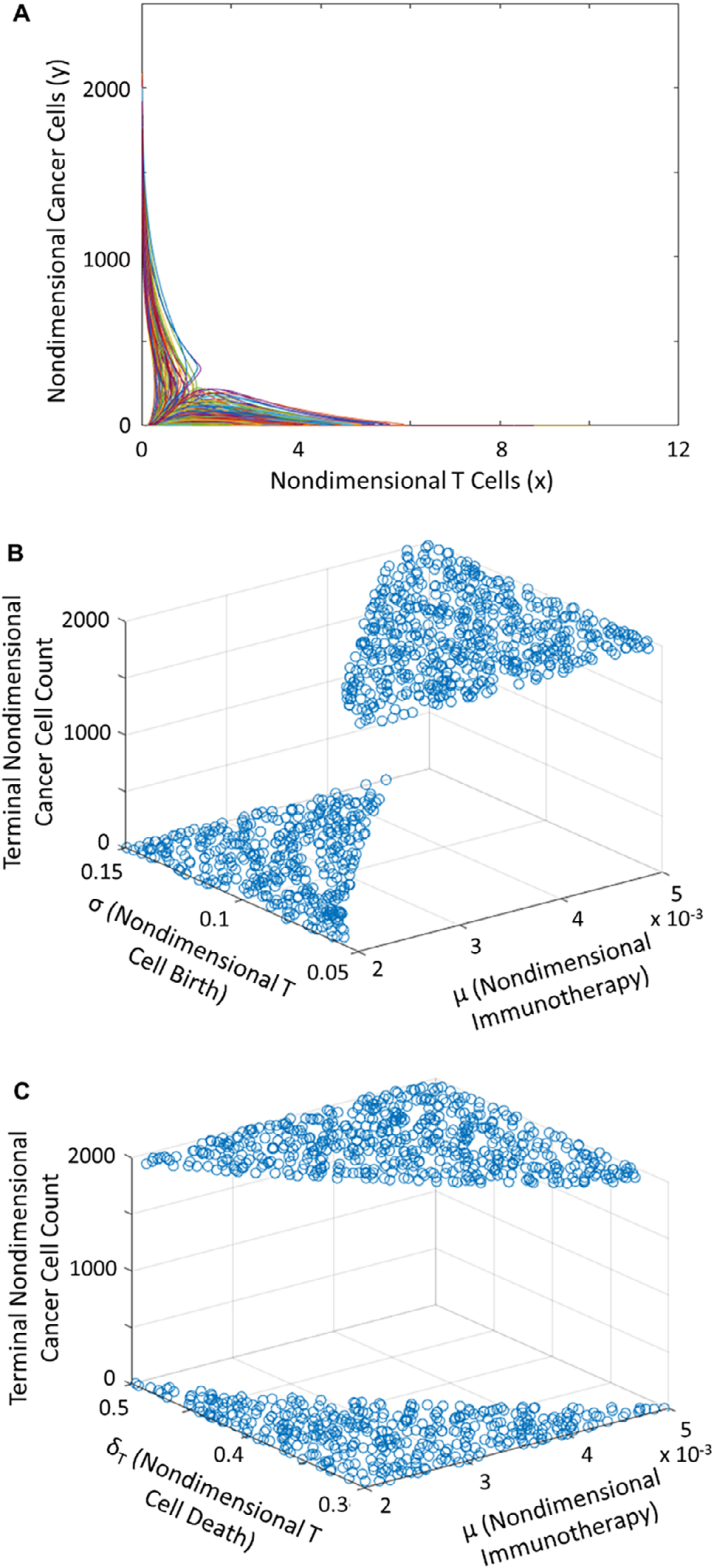
Nondimensionalized phase and parameter space for ODE system. (A) Phase plane representing nondimensionalized cancer cell vs. T cell count in the simulated tumor (*n* = 1000 runs). (B) Surface representing the effect of varying the cell birth‐immunotherapy parameter subset on cancer cells as an outcome. (C) Surface representing the effect of varying the cell death‐immunotherapy parameter subset on cancer cells as an outcome.

We confirmed that the nondimensional phase plane exhibited identical behavior (Figure 3A). To ascertain sensitivity of the terminal cancer cell population to immune checkpoint blockade, and other parameters’ contributions to the system, we performed a sensitivity analysis. A PRCC analysis supported the sensitivity of the terminal cancer cell population to the immune checkpoint term. However, due to parameter space nonlinearity and non‐convexity, we locally explored parameter subsets. We observed a clear delineation in the spaces spanned by (1) cell birth and immune checkpoint blockade (Figure 3B) and (2) cell death and immune checkpoint blockade (Figure 3C). These plateaus corresponded to progressive disease and stable disease steady states. The line of transition between these states suggested that immune checkpoint blockade compensated for lower T cell populations due to either low birth/recruitment or high death/apoptosis. This underscored the necessity of maintaining a critical threshold of active T cells in the tumor microenvironment.

### 3.2 ABM Results and Discussion

**FIGURE 4:**
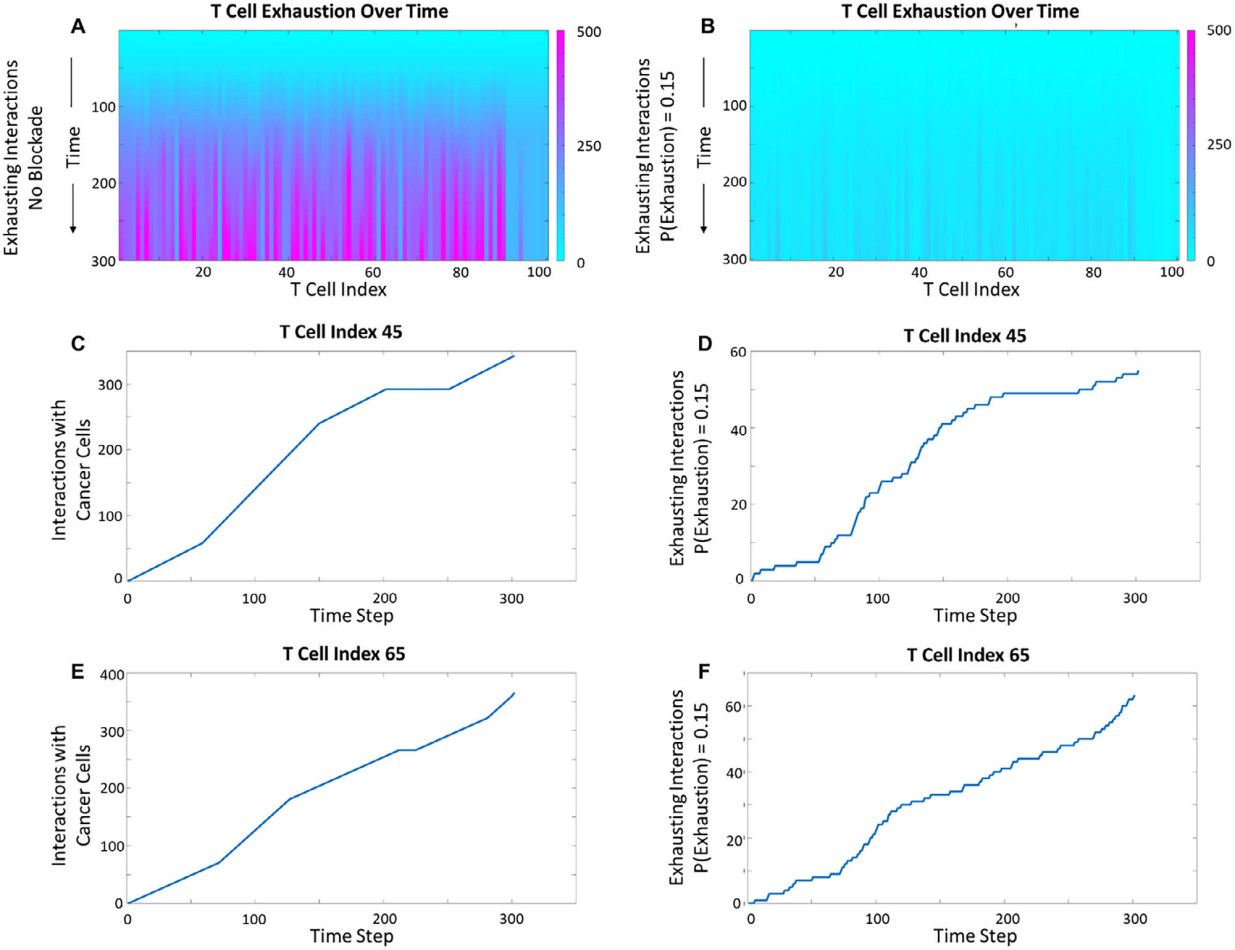
Representation of infiltrating T cell exhaustion trajectories in a subset of the solid tumor microenvironment. Color bar indicates the number of interactions (A) and predicted number of exhausting interactions in the presence of an 85% effective blockade (B). The total interactions (C, E) and expected exhaustion trajectory (D, F) are shown for representative T cells, indexed 45 and 65, in the center of the tumor. Representative T cells were selected from the cells that (i) had successfully infiltrated the tumor and (ii) were among neither the most nor least interactive cells throughout the duration of the simulation.

**FIGURE 5:**
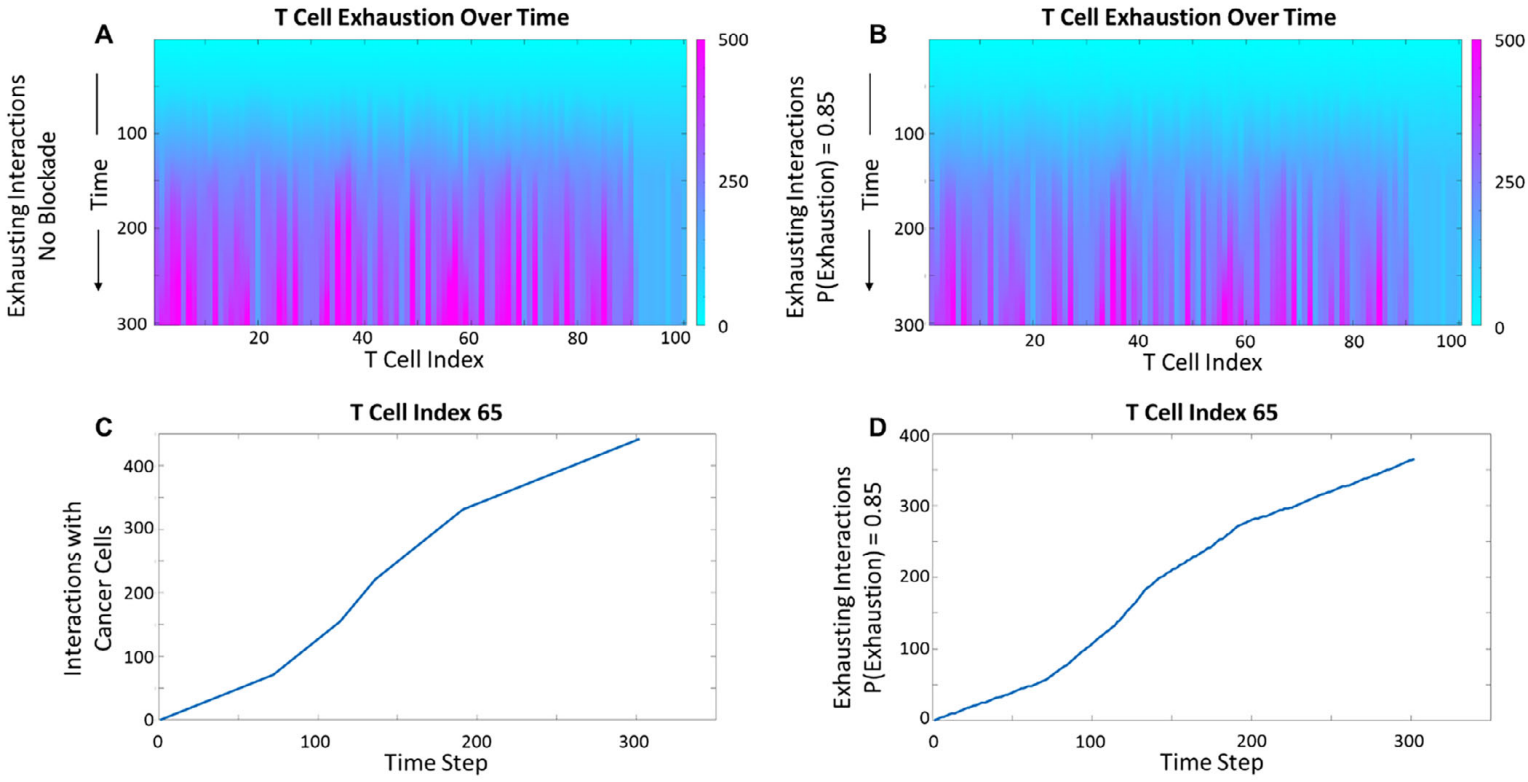
Representation of infiltrating T cell exhaustion trajectories in a subset of the solid tumor microenvironment. Color bar indicates the number of interactions (A) and predicted number of exhausting interactions in the presence of a 15% effective blockade (B). The total interactions (C) and expected exhaustion trajectory (D) are shown for a representative T cell, indexed 65, in the center of the tumor.

We simulated an 85% blockade strategy, consistent with the dynamical system predictions (Figure 4A, B). While nondimensional time units were used in this simulation, the behavior of initial T cell infiltration and expression of an exhaustion phenotype has been shown to occur within as early as 6 h [41]. At the individual cell level, phases of rapidly increasing interactions followed by lulls in activity appeared as multiple sigmoidal curves (Figure 4C–F). T cells infiltrating the tumor centrally encountered more cancer cells, yielding greater exhaustion scores. We repeated our sweep through percent blockade by imposing probabilities of T cell exhaustion given an interaction. The trends in systemic and individual cell exhaustion were preserved with some variability due to model stochasticity. As illustrated in Figure 5, the inverse of our blockade strategy (15% blockade) resembled the control case (no blockade).

We observed simulated solid tumors consistently expanding over time, given open boundary conditions. Tumor expansion due to mechanical forces, in addition to cell replication, is interpreted as a factor in metastasis [39]. Thus, future work exploring metastatic potential would be valuable.

Our future research includes iterations of this model that benefit from assuming a nonuniform or dynamic distribution of therapy in the tumor microenvironment, including a nonlinear exhaustion function, accounting for reversibility of exhaustion prior to a defined terminal exhaustion threshold, defining T cell “attraction” to recapitulate immune pockets seen in histology, and scaling the number of agents (cells) to represent a larger tumor. While current ABM simulations (on the order of 10^3^ cells and time scale of hours) are computationally efficient and reveal behaviors on the scale of a local cellular neighborhood, scaling to the order of 10^6^ cells and incorporating birth, death, and differentiation processes over longer times would allow the option for direct comparison with the ODE model. This is not only a valuable exercise in cross‐model validation; in addition to the new insights available at the whole tumor level, including the identification of emergent patterns or cell clustering behaviors, scaling and directly comparing the ABM and ODE models would provide further opportunities to integrate these complementary model systems. For example, a future combined model could rely more heavily on the ABM for specific cell‐level computations, while the ODE system could advance the model more efficiently at the tumor level, leveraging the difference in resolution. Comparing expanded model predictions between scales would reveal which cell‐level behaviors are dictated by local interactions and are most liable to be overlooked systemically.

Expanding the complementary ODE‐ABM model system is an advantageous avenue for future work in testing alternative assumptions regarding cellular activation and exhaustion trajectories, drug‐specific features, and patient‐specific features. As the current model assumes collective blockade of exhausting interactions, pathway‐ and tumor type‐specificity would be valuable delineations for future translational impact. For example, PD‐1, CTLA‐4, and other inhibitory receptors work via unique molecular mechanisms and feedback loops. Accounting for these pathway differences, and the pharmacokinetics of immune checkpoint blockade drugs, would be valuable for in silico testing of specific standard‐of‐care monotherapies and combination therapies. This work holds promise for testing strategies in virtual tumor and patient populations, yielding an invaluable framework for efforts in therapeutic engineering.

## 4 Conclusions

Using a combination of models at the whole‐tumor and individual cell levels, we have demonstrated predictions for a minimum desired immune checkpoint blockade efficacy such that stable disease becomes the most likely outcome. Our analyses furthermore (1) suggest that the terminal cancer cell population is sensitive to this blockade efficacy and (2) allow for the observation of individual T cell‐level exhaustion trajectories as an effect of imposed blockades.

## Author Contributions

AMT: conceptualization, methodology, formal analysis, writing—original draft. AJK: supervision, methodology, writing—review and editing. AMT and AJK read and approved the final manuscript.

## Conflicts of Interest

The authors have no conflict of interest to disclose.

## Availability of Codes

The codes used during the current study are available from the corresponding author on reasonable request.

## Disclaimer

Certain equipment, instruments, software, or materials are identified in this paper in order to specify the experimental procedure adequately. Such identification is not intended to imply recommendation or endorsement of any product or service by NIST, nor is it intended to imply that the materials or equipment identified are necessarily the best available for the purpose.

## Data Availability

The datasets used and/or analyzed during the current study are available from the corresponding author on reasonable request.
